# Design of Lidar Data Acquisition and Control System in High Repetition Rate and Photon-Counting Mode: Providing Testing for Space-Borne Lidar

**DOI:** 10.3390/s22103706

**Published:** 2022-05-12

**Authors:** Liangliang Cheng, Chenbo Xie, Ming Zhao, Lu Li, Hao Yang, Zhiyuan Fang, Jianfeng Chen, Dong Liu, Yingjian Wang

**Affiliations:** 1 Key Laboratory of Atmospheric Optics, Anhui Institute of Optics and Fine Mechanics, Hefei Institute of Physical Science, Chinese Academy of Sciences, Hefei 230031, China; clliang@mail.ustc.edu.cn (L.C.); zhaom@aiofm.ac.cn (M.Z.); lilu201205@163.com (L.L.); yh9599@mail.ustc.edu.cn (H.Y.); fangzhy@mail.ustc.edu.cn (Z.F.); jianfengchen1212@mail.ustc.edu.cn (J.C.); dliu@aiofm.ac.cn (D.L.); wyj@aiofm.ac.cn (Y.W.); 2 Science Island Branch of Graduate School, University of Science and Technology of China, Hefei 230026, China; 3 Advanced Laser Technology Laboratory of Anhui Province, Hefei 230037, China; 4 Anhui Province Key Laboratory of Simulation and Design for Electronic Information System, Hefei Normal University, Hefei 230601, China; 5 Anhui Province Key Laboratory of Target Recognition and Feature Extraction, Hefei 237000, China

**Keywords:** data acquisition, lidar control, SoC FPGA, photon-counting, space-borne lidar

## Abstract

For ground-based lidars in atmospheric observation, their data acquisition unit and control unit usually work independently. They usually require the cooperation of large-volume, high-power-consumption Industrial Personal Computer (IPC). However, the space-borne lidar has high requirements on the stability and integration of the acquisition control system. In this paper, a new data acquisition and lidar control system (DALCS) was developed based on System-on-Chip Field-Programmable Gate Array (SoC FPGA) technology. It can be used in lidar systems with high repetition rate and photon-counting mode and has functions such as data storage, laser control, automatic collimation, wireless communication, and fault self-test. DALCS has two working modes: in online mode, the echo data collected by DALCS are transmitted to the computer for display in real-time and then stored with the current time as the file name; in offline mode, the data are stored in local non-volatile memory, which can be read remotely and can work autonomously when there is no IPC. The test results showed that in the frequency range of 0–70 M, the counting linearity of DALCS reached 0.9999, and the maximum relative error between the DALCS card and the standard signal source was 0.211%. The comparison results showed that the correlation coefficient between DALCS and MCS-PCI was as high as 0.99768. The DALCS was placed in a self-developed lidar sensor system for continuous observation, and the system worked stably under different weather conditions. The range-squared-corrected signal profiles obtained from the observations reflect the spatial and temporal distribution characteristics of aerosols and clouds well. This provides scheme verification and experimental support for the development of space-borne lidar data acquisition and control system.

## 1. Introduction

Lidar uses a laser as a medium. The laser interacts with particles or various gas molecules in the atmosphere, and the backscattered echo signals are collected, which are eventually used to obtain atmospheric parameters through data inversion and analysis [1,2]. Lidar is widely used to detect atmospheric aerosols, water vapors, ozone, or temperature due to its high spatial and temporal resolution and high detection accuracy [3,4,5]. It mainly consists of a laser transmitter unit, an optical receiver unit, an acquisition unit, and a control unit [6,7], of which the acquisition unit and the control unit are important components [8]. The echo light signal is converted to an electrical signal by photoelectric sensors, such as Avalanche photodiode (APD) or Photomultiplier tube (PMT). The signal is then sent to the control unit after data acquisition and pre-processing. The control unit receives and stores the echo data, monitors the operating status of the laser and other components in real-time, sets the acquisition card parameters, configures the photodetector gain and high voltage, etc. It generally uses a universal Industrial Personal Computer (IPC) to achieve this, so that the lidar works in an orderly manner according to the set steps.

Depending on the platform, lidar can be classified as ground-based, mobile, airborne, and space-borne lidar [9]. They have different requirements for the acquisition and control system. For example, ground-based lidar has relatively low requirements. Commercial data acquisition cards are usually used to work with the IPC (control unit) to obtain higher spatial resolution. The acquisition and control system is generally not dedicated, which makes the ground-based lidar acquisition and control system relatively large. The requirements of space-borne lidar are much more complicated compared to ground-based lidar. For example, in the space environment, it is powered by solar energy and cannot be manually adjusted or maintained during orbital operation. Therefore, the electronic system is required to have high stability and integration. Due to the limitation of size, power consumption, stability, and other factors, the software and hardware architecture of the lidar data acquisition and control system is different from that of ground-based lidar, and there is no such mature and available equipment in the market. At present, the representative space-borne lidar in the cloud and aerosol detections are CALIOP (Cloud-Aerosol Lidar with Orthogonal Polarization) and CATS (Cloud Aerosol Transport System). CALIOP is a space-borne lidar launched by NASA in 2006. In the aspect of control, it measures the detector’s dark current through the movable shutter and controls the depolarizer to enter the 532 nm channel for depolarization calibration [6,10,11,12]. It not only monitors the working status of the laser (e.g., laser energy) but also monitors other components (acquisition card, controller, drive motor, power supply) to ensure reliable operation of the system. Through the pre-processing of the data acquisition and control system, it generates different levels of data products for researchers to download. CALIOP acquires the echo signal using analog detection mode due to the large energy of the single pulse of the laser it uses (110 mJ) [10]. While CATS is the first space-borne lidar using photon-counting mode, which has two lasers with a single pulse energy of 1 mJ/2 mJ and a repetition rate of 5 kHz/4 kHz, respectively. CATS increases the average transmit power of the laser by increasing the pulse repetition frequency. In this detection mode, the echo signal received by the lidar is extremely weak, only in the order of photons. If the analog detection mode is used, the effective signal will not be extracted from the background noise. Therefore, photon-counting technology was used. The intensity of the echo signal at different distances is obtained statistically, which puts higher requirements on data acquisition. In terms of control, it realizes whether to generate the 355 nm band by controlling the moving mirror, leading to and away from the Third Harmonic Generation (THG), which validated the feasibility of using the 355 nm band for space-borne lidar to detect the atmospheric environment [13,14,15].

Previous reports show that researchers have designed photon-counting cards (multi-channel scaler: MCS) based on high-speed digital chips, which have a complex circuit structure. Marek Zieliński et al. designed a real-time multichannel scaler with a high time resolution of 5 ns, but it supports a low laser repetition rate of 50 Hz (at 2^15^ time channels) [16,17]. With the development of semiconductor technology, field-programmable gate array (FPGA) technology with high-speed parallel processing advantages has attracted more attention from researchers. Z. Guzik et al. designed the TUKAN data acquisition system, which has multi-channel analysis and multi-channel scaling function, but the maximum counting rate of MCS is only 8 MHz [18,19], so it does not support photon-counting detectors used by lidar. Commercially, available and mature photon-counting cards are MCS-PCI and EASY-MCS, which use Peripheral Component Interconnect (PCI) and Universal Serial Bus (USB) bus to communicate with a computer, respectively. They use sophisticated digital circuits to eliminate the effect of dead time caused by channel switching. It is widely used in biological, chemical, atmospheric, and environmental fields [20,21]. However, the data triggered each time needs to be uploaded to the computer for software accumulation, the software provided does not support continuous acquisition. They have a maximum vertical resolution of 15 m (minimum bins width is 100 ns), so they are not dedicated for lidar detection in the atmospheric field. AMCS-USB is used in NASA GSFC lidars such as Holographic Airborne Rotating Lidar (HARLIE), which has a vertical resolution of up to 7.5 m. However, at this resolution, the detection range is only nearly 30 km (the maximum number of bins is 4 K) [22]. Due to the limitation of internal circuit structure and transmission rate (USB 1.1 interface), it is not suitable for high repetition rate space-borne lidar. The Licel transient recorder supports both analog and photon-counting modes, it transmits data with Ethernet interface [23], but it is relatively large due to its modular structure. All of the above only has a data acquisition function, while data transmission, data storage, and control of lidar components need the participation of IPC, which increases the complexity of the whole lidar structure. Analysis of technical parameters of space-borne lidar are shown in Table 1. Based on previous reports and research, we believe that the space-borne lidar in high repetition rate and photon-counting mode is suitable for remote sensing monitoring of regional haze pollution, so we have carried out the development of the current lidar data acquisition and control system.

This paper proposes a software-hardware co-design method based on System-on-Chip (SoC) FPGA (Xilinx Zynq7020), and we develop a system (DALCS) integrating data acquisition and lidar control function, which supports triggering the high repetition rate signal, photon counting, and the control of lidar components. Zynq is a heterogeneous multi-core chip with integrated FPGA and Advanced Reduced Instruction Set Computer Machine (ARM), where the functions related to echo photon signal acquisition are implemented in the FPGA, including segment counting, accumulation, and data transmission of dual-channel photon signals. In addition, background noise removal [24] algorithm, laser status monitoring, data storage, wireless communication, automatic collimation, and other functions are executed in ARM. The second part of the article introduces the system structure and key parameters of the self-developed lidar, which works in high repetition rate and photon-counting mode, in which the laser, the mirror adjustment frame, and other components need to work under the control of DALCS.

## 2. Data and Methods

### 2.1. Lidar Sensor System

**Figure 1:**
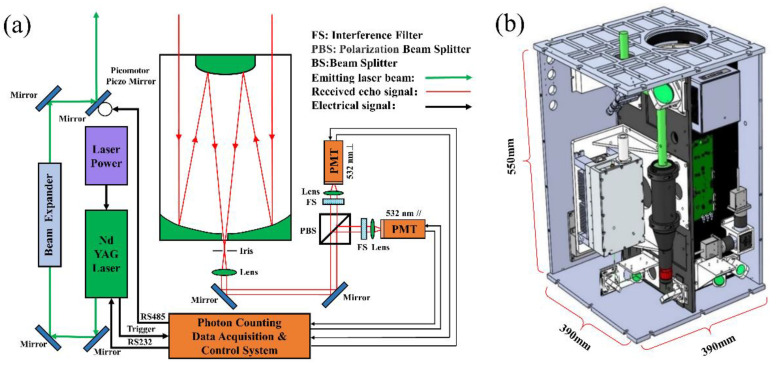
Lidar sensor system: (**a**) schematic diagram and (**b**) internal structure diagram.

DALCS is an important part of the lidar sensor system, which was developed by the Key Laboratory of Atmospheric Optics Center of Anhui Institute of Optics and Mechanics, Chinese Academy of Sciences. It is placed in Science Island, Hefei, Anhui Province, China (Longitude: 117.175, Latitude: 31.907). Figure 1 shows the principle and internal structure of the lidar sensor system. The transmitter system uses an Nd:YAG high repetition rate pulsed laser. Under the control of DALCS, it emits a 532 nm laser and simultaneously outputs a trigger signal. The direction of the laser is adjusted by the reflector, and the laser divergence angle is compressed by the beam expander and finally emitted into the atmosphere. The backscattered echo signal is received by the Cassegrain telescope. The small aperture on the back focal plane of the telescope limits the receiving field of view, which can reduce the sky background noise. The collimating lens further converts the echo light signal into parallel light. Then, the light is guided into the polarizing prism by the reflector, so that the echo light signal is divided into horizontal and vertical detection channels, and filtered by a narrow-band interference filter. Then, the lens focuses the parallel light onto the target surface of the PMT sensor, which has a photon detection ability. DALCS collects echo photon signals in real-time under the synchronous trigger signal. The reflector at the last stage of the transmitting unit adopts a 2-inch picomotor piezoelectric reflector adjustment frame (including X and Y axes). When the direction of the emitted laser beam deviates from the receiving field of view of the telescope, DALCS can adjust the reflection angle of the reflector to make the receiving and emitting paths parallel. In addition, to ensure the stable operation of the lidar sensor system, DALCS can monitor the working state of each lidar component and obtain the system operation and environmental state parameters.

The detailed parameters of the lidar sensor system are shown in Table 2. Due to the size limitation of the satellite payload platform, a small (340 mm × 120 mm × 125 mm), high repetition rate laser was selected. The pulse repetition frequency of the laser is 3 kHz, and the single pulse energy is 1 mJ. After the laser is expanded by 20 times, the divergence angle is compressed to 113 μrad, which ensures a longer detection distance. As the core component of the receiving optical system, the telescope also adopts a miniaturized design with a diameter of 125 mm, a field of view angle of 280 μrad, and a diaphragm aperture of 0.5 mm. The data acquisition and control unit adopts photon-counting detection mode. The pulse width of the PMT detection module output is 10 ns, and the pulse pair resolution is 20 ns. Bins width corresponds to the distance resolution, e.g., 100 ns corresponds to 15 m (this can be calculated from the speed of light propagation in the atmosphere). The number of bins is the number of acquisition points, and its product with bins width determines the detection range. The amount of accumulation is limited by the cumulative sum (not to exceed 4 M). Three adjustable parameters can meet the detection requirements of lidar in different application environments.

The backscattered echo signal received by lidar can be given by the lidar equation [6,9]:

(1) $$P(z)=\frac{1}{z^{2}}K\beta (z)\exp\left\{-2\int_{0}^{z}\alpha (z^{\prime })dz^{\prime }\right\}$$

where *P*(*z*) represents the echo signal power (unit: W) received by the lidar at the height *z*. Since the echo signal power is inversely proportional to the square of the detection range *z*, we usually use the range-corrected signal (*P*(*z*) × *z*^2^) to reflect the spatiotemporal distribution characteristics of aerosols and clouds; K is a system constant, which is related to the lidar structure (unit: W × km^3^ × sr); *β*(*z*) = *β*_a_(*z*) + *β*_m_(*z*), where *β*_a_(*z*) is the backscattering coefficient of aerosol at height *z* (unit: km^−1^ × sr ^−1^), *β*_m_(*z*) is the backscattering coefficient of atmospheric molecules; α(*z*) = α_a_(*z*) + α_m_(*z*), where α_a_(*z*) is the extinction coefficient of the aerosol (unit: km^−1^), and α_m_(*z*) is the extinction coefficient of the atmospheric molecule. $\exp\left\{-2\int_{0}^{z}\alpha (z^{\prime })dz^{\prime }\right\}$ is the transmittance of laser to and from the atmosphere, which reflects the attenuation effect of aerosol and molecules on the laser when the laser enters the atmosphere. The horizontally and vertically polarized signals collected by DALCS can be used to retrieve the extinction coefficients of atmospheric aerosols and clouds. Common methods are Slope method [25], Fernald method [26], and Klett method [27].

### 2.2. Zynq-Based Acquisition and Control System

(1) Hardware design

**Figure 2:**
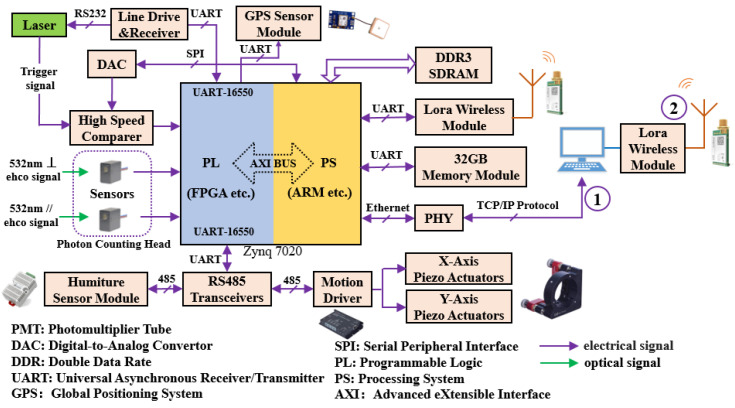
Block diagram of DALCS hardware structure.

Figure 2 is a block diagram of the hardware structure. ZYNQ-7020 integrated a processing system (PS) and programmable logic (PL) unit. PS is a processing system that integrates ARM Cortex-A9 core, and PL has programmable logic resources, which can be used as peripherals of ARM [28,29]. In the DALCS of this paper, the pulse signal counting and acquisition function was implemented in FPGA, and the programs related to device control were run in ARM. Zynq-7020 is connected with lidar components (such as laser, memory module, wireless module, electric adjustment rack) through UART, SPI, Ethernet, and other communication interfaces. For example, the PS sends commands to the DAC TLV5612 [30] (Texas Instruments, Dallas, TX, USA). Through the SPI bus, and the DAC output voltage is used as the reference voltage for the high-speed comparator ADCMP600 [31] (Analog Devices, Norwood, MA, USA). Since only two UART modules were integrated into the PS, an IP core (Axi uart16550 [32]) was added to the PL to expand the serial communication function, which was used to connect the laser and motion driver. DALCS has both online and offline modes (marked in Figure 2). In the online mode, the data are sent to the host computer through the Ethernet, which has good real-time performance and can be used for echo signal detection of ground-based lidar. In offline mode, the data are stored in non-volatile memory and sent to a remote computer wirelessly, which is suitable for use in special environments such as space-borne lidar.

(2) Principle and logical realization of photon-counting

**Figure 3:**
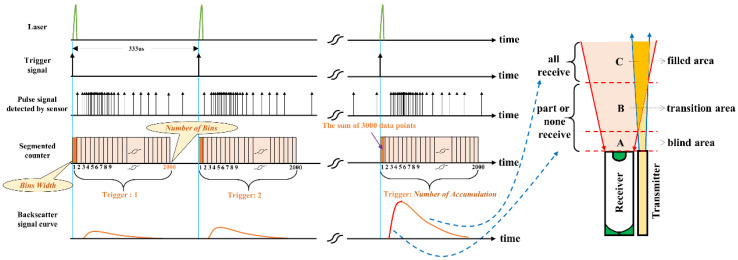
Block Schematic diagram of echo photon signal acquisition.

The echo photon signal acquisition process of the lidar sensor system is shown in Figure 3. When the laser emits the laser, it outputs a trigger signal synchronized with the laser. After being discriminated by the comparator, the signal is used as the starting signal of sampling. The echo signal is detected by the PMT module and then input to the acquisition unit. To obtain the distribution characteristics of aerosol concentration at different heights, the segmented counting method is used to count the echo photon signal, which is based on FPGA technology [33]. Due to the existence of shot noise, electronic thermal noise, the background light of the sky will also cause the detector to output electronic pulses, and “dark noise” will cause “dark counts”, which has a great impact on the observation accuracy of lidar. In this paper, the echo signals of the same height interval (bins width) are correspondingly accumulated to improve the signal-to-noise ratio [34]. Due to the influence of the blind area (mark A) or the transition area (mark B), the telescope cannot receive or partially receive the echo signal. In the filled area (mark C), the backscattered signal is fully received by the telescope. Therefore, the detected echo intensity curve shows a trend of first rising and then falling.

**Figure 4:**
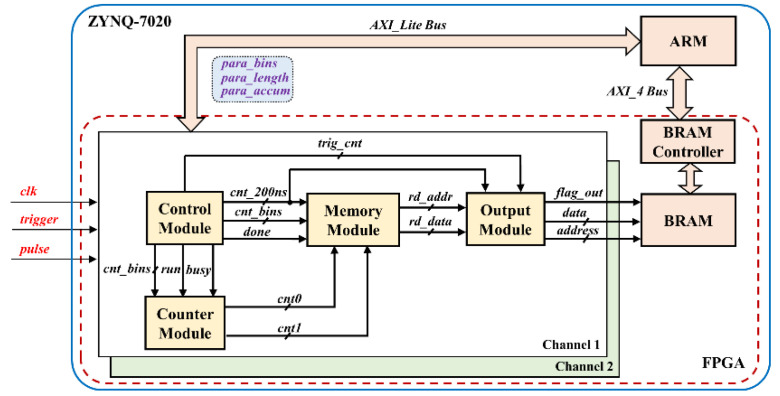
The Overall logical structure of the acquisition system.

The echo photon signal acquisition system is implemented in FPGA, and the overall logic structure is shown in Figure 4. The system has two channels to collect the backscattered signal’s parallel and perpendicular polarization parts. Each channel includes a control module, counting module, storage module, and output module. By receiving the trigger signal output by the laser as the enable signal, the acquisition, counting, and storage of the photon signal are completed. After the counting of the specified specifications is completed, the final collected data are sent to the data buffer (Block Read Only Memory (BRAM), BRAM controller) through the output module to complete the data transmission between FPGA and ARM. In addition, the ARM part can also change the time channel (para_bins), the sampling length (para_length), and the number of accumulations (para_accum) through the AXI_Lite bus. This enables the dynamic configuration of the FPGA count specification.

**Figure 5:**
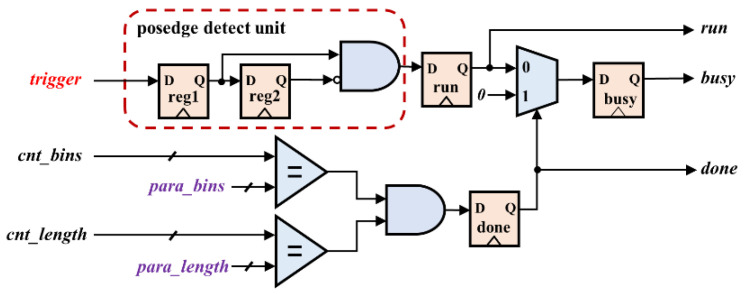
The control module logic structure diagram.

The control module monitors the entire acquisition process, as shown in Figure 5. This module mainly completes the real-time control of counting, storage, and output modules by generating run, busy, and done signals. The rising edge detection of the trigger is completed by processing the register beat of the external trigger signal. When the rising edge of the trigger signal comes and done is low, the run is pulled high, indicating that the single round counting of the acquisition system begins to work. When the run is high and done is low, busy is pulled high, indicating that the single round counting of the acquisition system is in progress. As the values of the internal registers cnt_bins and cnt_length are equal to the set values of para_bins and para_length, respectively, done is pulled high, and the single-round counting work of the acquisition system ends.

**Figure 6:**
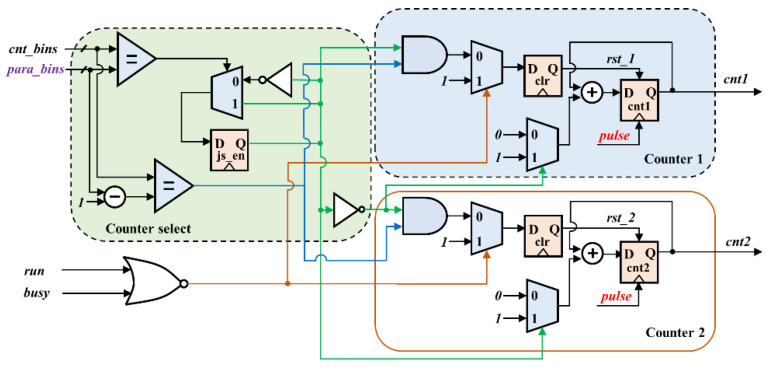
The logic structure diagram of the counting module.

The counter module is the core component of the acquisition system, as shown in Figure 6. The module is designed with asynchronous logic, where registers (cnt1 and cnt2) are driven by random pulses from an external echo photon signal. First, a square wave signal (js_en) with a period of 2× para_bins is generated by counter select to enable cnt1 and cnt2 to count alternately with para_bins as the time unit. As js_en is pulled high and the values of the internal registers (cnt_bins and para_bins-1) are equal, the clear signal rst_1 is pulled high, and rst_1 is 0 in other cases. Conversely, when js_en is pulled low and the values of the internal registers (cnt_bins and para_bins-1) are equal, the clear signal rst_2 is pulled high, and rst_2 is 0 in other cases. The rst_1 and rst_2 perform clearing of registers cnt1 and cnt2, respectively. The values of registers cnt1 and cnt2 are increased by 1 for each random pulse of the external echo photon signal received, as long as rst_1 and rst_2 are not pulled high. This method of using two registers to count alternately can effectively reduce the probability of external pulse loss.

**Figure 7:**
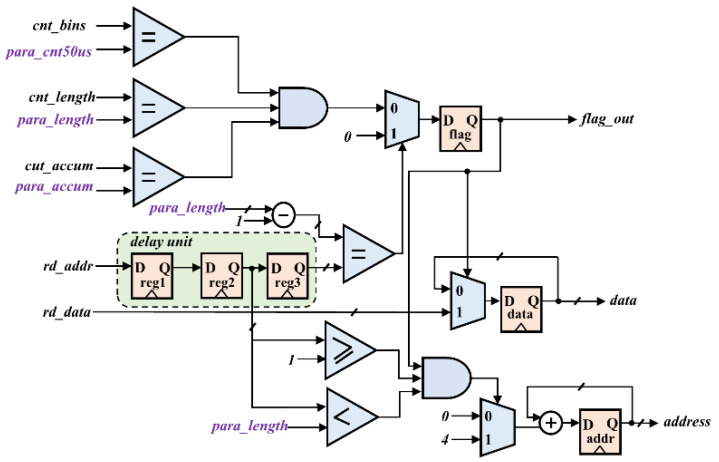
The logic structure diagram of the output module.

The final result of the sampling system count needs to be output to the ARM for pre-processing. Figure 7 shows the logic of the data output module. The output module writes the count results to the BRAM memory in the order of acquisition, and then the BRAM controller transmits them to the Double Data Rate 3 Synchronous Dynamic Random-Access Memory (DDR3 SDRAM) via the high-performance Advanced eXtensible Interface 4 (AXI4) bus. After data transmission is completed, FPGA notifies the ARM processor through an interrupt. ARM is connected to SDRAM, so it can quickly read out data, increasing the timeliness. The flag_out is a transmission status signal, and data transmission starts when flag_out is high. The following conditions for transmitting data must be satisfied: the bins countercnt_bins, the sampling length counter cnt_length, and the accumulative times counter cnt_accum are respectively equal to the parameters transmitted from the ARM. The sampled data (data) are sequentially transferred to the BRAM under the synchronization of the address(addr). Since the width of the AXI data bus is 32 bits, the addr signal is increased by 4 each time.

**Figure 8:**
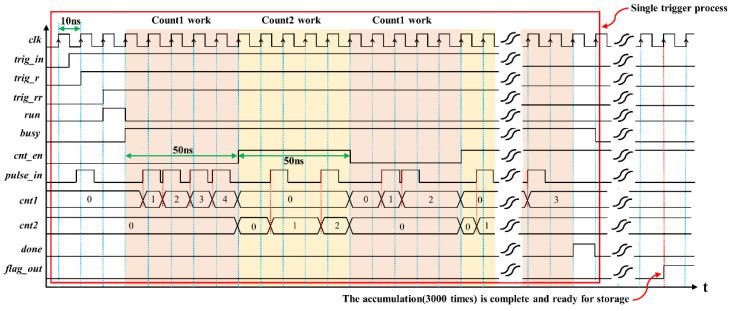
Main logic signal diagram of the acquisition system.

The timing diagram of the main logic signals is shown in Figure 8. The main clock signal (clk) of the acquisition system is generated by a crystal oscillator and a phase-locked loop (PLL). The red box in Figure 8 is a single trigger process, and flag_out is the output flag signal after accumulation. After the accumulation (3000 accumulations here), the data will be output to ARM. After the external trigger signal trig_in is input to the acquisition system, the run signal is output by the rising edge detection circuit. Run is the counting start signal; when it has a falling edge, the busy signal is pulled up, indicating that the counter is working. Once the count is completed, done outputs a high-level signal for one cycle, indicating the end of sampling caused by a single trigger. To reduce the influence of counting dead time, two counters are used for time-sharing, that is, when one of the counters counts, the other is stored. The cnt1 and cnt2 are two independent counters, and the length of bins (50 ns here) in a single counting cycle can be adjusted. Cnt_en selects these two counters to prevent missing count events caused by data storage procedures. This paper adopts the design method of asynchronous logic, which can effectively reduce the probability of missing sampling.

(3) Software design

**Figure 9:**
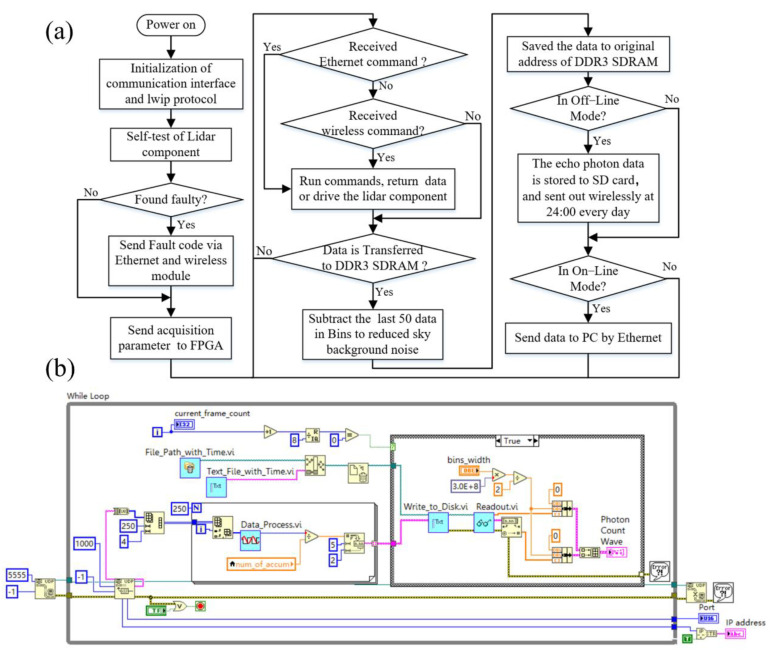
Software design: (**a**) embedded program flow chart; (**b**) the main program of the upper computer.

The embedded program is designed based on Xilinx SDK 2017.4 software by Xilinx (San Jose, CA, USA) software and developed in C language. Figure 9a shows the software flow chart. The program runs on the ARM side of the Zynq chip, including device initialization, system status self-check, echo data pre-processing, local storage, and network transmission. (1) After the acquisition control system is powered on, the control program first initializes the peripherals, acquisition cards, and communication protocols, then performs self-checks on key components such as lasers, and reads the device status information. (2) After the self-test is completed, ARM sends commands to the laser through the serial port to make it emit laser light. Then, ARM sends three acquisition parameters (bin_width, length, and accumulation) to FPGA through the AXI_Lite bus to make it work. (3) After receiving the interrupt request from FPGA, ARM immediately reads data from DDR3 SDRAM and executes the background noise removal algorithm to calculate the read echo data, which is finally stored in the buffer. (4) In online mode, DALCS sends data to the computer via Ethernet. The data acquisition and control software of the computer reads the data in real-time and stores it in the hard disk according to the preset format. (5) In offline mode, DALCS runs automatically, the data are stored in non-volatile memory after pre-processing and are periodically sent to the computer wirelessly.

In online mode, the main program of the upper computer software is shown in Figure 9b. The software is designed based on LabVIEW. It contains lidar component control, data receiving, data processing, waveform display, and data storage functions. Firstly, the computer sends the acquisition parameters to the DALCS card and monitors the working status of key components, such as laser working current and temperature. Secondly, when receiving the data from the acquisition card, the computer displays it in the waveform chart after processing (data reorganization and linear correction are realized in data_process.vi). Finally, the data are stored in ASCII format in ‘.txt’ files named ‘hour-minute-second’ (e.g., ‘081902’), while the daily data are stored in a folder named ‘year-month-day’ (e.g., ‘20220301’), which is used to generate the spatial-temporal distribution of the aerosols by inversion. In offline mode, DALCS runs an automatic control program. It can also be manually intervened by the upper computer software wirelessly, such as obtaining the working state of lidar components. Its data processing mode is similar to the online mode, but the communication mode is a serial port (connecting the wireless communication module).

## 3. Results and Analysis

### 3.1. Data Acquisition Testing and Analysis

**Figure 10:**
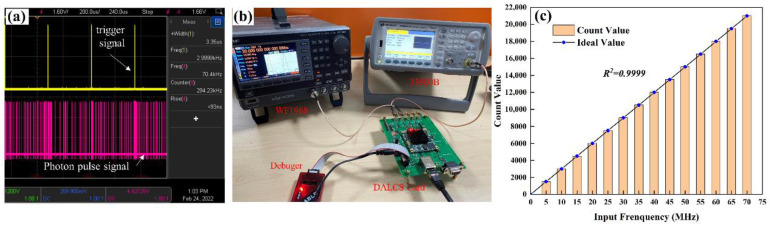
Signal test: (**a**) Laser and PMT module output signal; (**b**) Test environment; (**c**) Count linearity.

To test the linearity of the acquisition and control system, according to the characteristics of the output signal of the laser and the PMT module (as shown in Figure 10a), two function generators are used to simulate the trigger signal and the photon signal, respectively (the two signal generators can reduce their correlation), and to test the count value output by DALCS under different pulse signal frequencies. The experimental process is as follows: signal generator 33500B (signal jitter <40 ps RMS) by Keysight (Santa Rosa, CA, USA) outputs a pulse signal (3 kHz frequency, 3.33 µs pulse width) to simulate the trigger signal (the same parameters as the laser output trigger signal); signal generator WF1968 (signal jitter 90 ps RMS) by NF (Kohoku-ku, Yokohama, Japan) outputs a narrow pulse signals of different frequencies (0–70 MHz). Set the width of the bins to 100 ns, the Number of Bins to 2000, and the Number of Accumulation to 3000. Count values were captured by Integrated Logic Analyzer (ILA) in Vivado software (Xilinx: San Jose, CA, USA). As can be seen in Figure 10c, the mean count value agrees well with the ideal value (black line) when different frequency pulse signals are input, and the correlation coefficient reaches 0.9999, and the maximum relative error between the DALCS card and the standard signal source was 0.211% (At an input frequency of 35 MHz). It can be seen from the test results that DALCS supports the triggering of 3 kHz high repetition frequency pulse signals, so it can be used for lidar with a high repetition rate laser.

### 3.2. Hardware and Software Collaborative Debug

**Figure 11:**
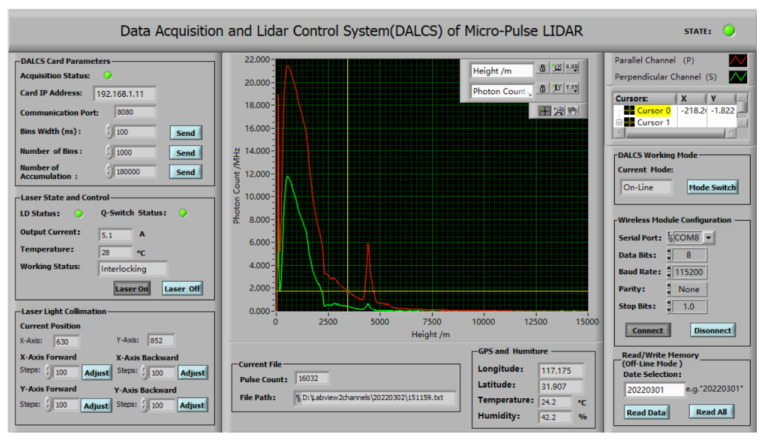
The upper computer software of DALCS.

To verify the stability and reliability of data transmission, data pre-processing, and lidar control functions, the echo signals were acquired by self-developed upper computer software (developed based on LabVIEW). As shown in Figure 11, both sides of the software are control functional areas, which mainly include the parameter setting or status monitoring of the DALCS card, laser, wireless communication module, and the laser collimation system. We installed the card in a lidar sensor system for continuous testing. In online mode, the laser is turned on by this software, and the DALCS card monitors the operational status of the lidar components (e.g., laser and collimation system) and the operational environment parameters (e.g., temperature and humidity and latitude and longitude), and the latter is sent out together with the collected photon-counting data. In offline mode, DALCS works autonomously. The DALCS card directly sends control commands to make the laser emit light. The sensor data, together with the photon-counting data, are stored in the non-volatile memory and sent to the terminal computer wirelessly for a fixed period (24 h). After debugging, the software can reliably send and receive data in both online (Ethernet) and offline (wireless) modes. The received data are pre-processed, stored in the local disk in turn with the time as the file name, and the echo photon signal of the two channels is displayed in the waveform chart. When a fault is monitored, the indicator in the upper right corner of the software turns red.

**Figure 12:**
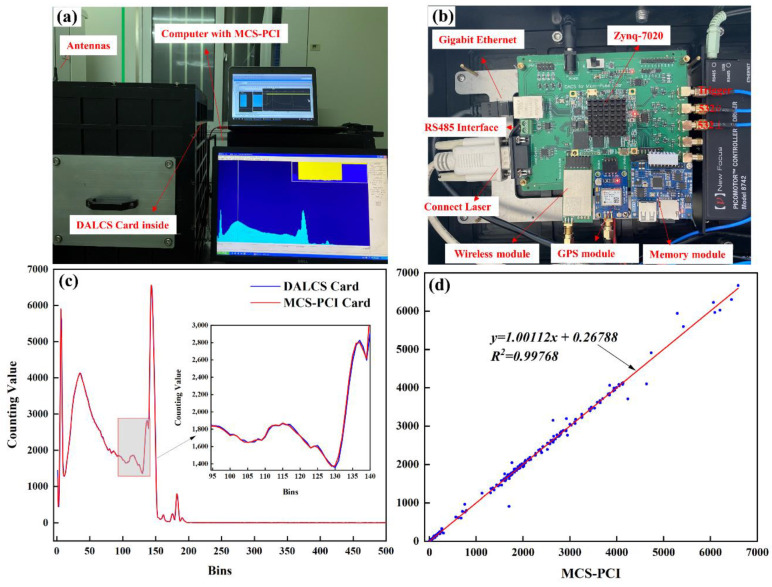
Actual test: (**a**) Lidar test platform; (**b**) DALCS Card; (**c**) comparison of D ALCS and MCS-PCI; (**d**) linear fitting of DALCS and MCS-PCI.

To further test the measurement accuracy of DALCS in lidar applications, the DALCS card (Figure 12b, size 131 × 96 mm) was installed on the lidar sensor system (the left side of Figure 12a) and compared with the MCS-PCI card. The frequency-divided trigger signal and the photon signal of the 532 nm parallel polarization channel are connected to the DALCS and MCS-PCI cards at the same time. Under the same parameters (bin width is 100 ns, number of bins is 1000, number of accumulation is 3000), the test results are shown in Figure 12c,d. When the signal changes, the DALCS and MCS-PCI count values match well in different bin intervals. It can be seen that DALCS has high counting accuracy and time resolution. The linear fit plots of the two are shown in Figure 12d, and the correlation coefficient is as high as 0.99768, which indicates that the two are in good agreement.

### 3.3. Application Synthesis Experiment and Analysis

**Figure 13:**
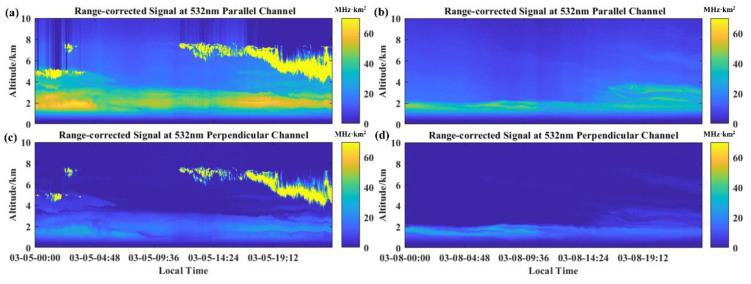
Range-corrected signal profiles in different weather: (**a**) 532 nm parallel Polarization channel on 5 March 2022; (**b**) 532 nm vertical Polarization channel on 5 March 2022; (**c**) 532 nm parallel Polarization channel on 8 March 2022; (**d**) 532 nm vertical Polarization channel on 8 March 2022.

The DALCS is placed in the lidar sensor system, and the 532 nm parallel and perpendicular polarization channel signals are continuously collected. To verify the adaptability of DALCS to different environments, different weathers were selected for comparison. Meteorological information shows that 5 March 2022 was cloudy with light pollution, and 8 March 2022 was sunny with good air quality. Therefore, the data detected by DALCS on these two days were selected for comparison. Figure 13a,c show the spatial and temporal distribution of the range-corrected signals of the 532 nm parallel and perpendicular polarization channels detected on 5 March, respectively. As seen in Figure 13a, clouds appeared at 5 km altitude from 0:00 a.m. to 4:00 a.m., including a temporary high cloud at 7.5 km at around 3:00 a.m., with a cloud height of nearly 8 km. There is a thicker aerosol layer at a low altitude of 1–3 km, with a maximum intensity of 70 MHz·km^2^, nearly 8 km. There was a thicker aerosol layer at 1–3 km at low altitude, with a maximum intensity of 70 MHz·km^2^. Around 12:00, clouds appeared at an altitude of 7.5 km, and at 8:00 p.m., the thickness of the cloud layers increased, and the cloud height began to decline. Figure 13b,d show the spatial and temporal distribution of the range-corrected signal detected on 8 March. As can be seen from Figure 13b, the sky was cloudless that day and only low concentrations of aerosols were present, with intensities mainly between 30 and 45 MHz·km^2^ and with aerosol stratification. Before 5 p.m., aerosols are mainly distributed below 2 km; after 5 p.m., aerosols are present below 4 km. The experiments confirm that the designed DALCS is capable of detecting the vertical optical properties of aerosols in clean, polluted, and cloudy weather with high spatial and temporal resolution. The control unit provides a guarantee for the collection and transmission of echo photon signals so that DALCS can work stably and continuously in different environments.

## 4. Conclusions

Based on SOC FPGA technology, a system integrating data acquisition and lidar control (DALC) was developed in this paper. Through testing and joint debugging on the self-developed lidar sensor system, we carried out continuous vertical observations. The conclusions are summarized as follows: (1) We have implemented the photon-counting function of echo signals on Zynq-7020. In the counting method, the asynchronous logic design was adopted to reduce the probability of missing photon pulse signals. The accumulation function was realized by hardware on FPGA, so it can support the triggering of a 3 kHz high repetition rate laser. In the frequency range of 0–70 MHz, the counting linearity of DALCS reaches 0.9999, and the maximum relative error with the standard signal source is 0.221%. Compared with the actual measurement, the linear correlation between DALCS and MCS-PCI is as high as 0.9983. (2) The echo signal acquisition and lidar control functions are integrated on a circuit board. The communication interface is extended on the FPGA, and the control program is executed on the ARM, which makes the DALCS have functions such as fault self-test, lidar component status monitoring, local storage, wireless communication, and auto-collimation. Through the software and hardware co-design of the electronic system, the DALC is integrated with the optical-mechanical structure of the lidar sensor system. In addition, the data collected by FPGA is transmitted to ARM through the internal AXI bus of the chip, which reduces the probability of external interference. These advantages meet the design requirements of high integration and high stability of space-borne lidar. (3) The range-square-corrected profiles obtained from lidar observations reflect the spatiotemporal distribution characteristics of aerosols and cloud particles. In a variety of weather environments such as clean weather, polluted weather, and cloudy weather, the DALCS can work stably in both online and offline modes. This DALCS has flexible functions, high integration, and good stability. It provides a new option for space-borne lidar with high repetition rate and photon-counting modes.

## Figures and Tables

**Table 1:** Analysis of technical parameters of space-borne lidar.

Parameter	MCS-PCI	EASY-MCS	AMCS-USB	Licel TR20-160
Minimum bins width	100 ns	100 ns	50 ns	50 ns
Maximum number of bins	64 K	64 K	4 K	32 K
Maximum number of accumulation	4 M-1	4 M-1	32 K	2 M-1
Wired communication interface	PCI	USB 2.0	USB 1.1	10/100 Ethernet
Control function (wireless, storage, self-test, collimation, etc.)	No	No	No	No
IPC is needed when working	Yes	Yes	Yes	Yes

**Table 2:** Main parameters of lidar.

Part	Parameters	Value	Part	Parameters	Value
Laser emitting unit	Laser wavelength/nm	532.18	Data acquisition and lidar control unit	Detector	PMT
	Divergence angle/μrad	113		Pulse width/ns	10
	Single pulse laser energy/mJ	1		Pulse-pair resolution/ns	20
	Pulse repetition rate/Hz	3 k		Acquisition mode	Photon-counting
	Line width/pm	45		Number of Channels	2
	Pulse width /ns	13		Maximum counting rate/MHz	250
Optical receiving unit	Telescope diameter/mm	125		Minimum bins width/ns	20
	Iris/mm	0.5		Maximum number of bins	4 M-1
	Field of view/μrad	280		Maximum number of accumulation	4 M-1
	Telescope focus distance/mm	1430		Data storage mode	Store or Sending
	Filter bandwidth/nm	0.3			

## Data Availability

Not applicable.
